# Comparison between erector spinae plane block and paravertebral block regarding postoperative analgesic consumption following breast surgery: a randomized controlled study

**DOI:** 10.1186/s12871-022-01724-3

**Published:** 2022-06-18

**Authors:** Ahmed M. Elewa, Mohammed Faisal, Folke Sjöberg, Mohamed E. Abuelnaga

**Affiliations:** 1 Department of anaesthesia, critical care and pain management, Faculty of Medicine, Suez Canal University, Ard Elgameiat, Ismailia, Egypt; 2 Department of Surgery, Faculty of Medicine, Suez Canal University, Ismailia, Egypt; 3 General Surgery Department, Sahlgrenska University Hospital, Gothenburg, Sweden; 4 Department of Biomedical and Clinical Sciences (BKV), Linköping University Hospital, Linköping, Sweden

**Keywords:** Erector spinae plane block, Modified radical mastectomy, Paravertebral block, Postoperative pain

## Abstract

**Background:**

Pain control following breast surgery is of utmost importance in order to reduce the chance of chronic pain development, and facilitate early rehabilitation. The erector spinae plane block (ESPB) is a recently developed regional anaesthesia procedure successfully used for different types of surgical procedures including thoracic and abdominal surgeries.

**Methods:**

A double-blind, randomized, controlled trial was conducted on 90 patients who were scheduled for modified radical mastectomy (MRM). Patients were randomly categorized into groups I (women who underwent ESPB), II (women who underwent paravertebral block (PVB), and III (women who underwent general anaesthesia).

**Results:**

The ESPB (4.9 ± 1.2 mg) and PVB (5.8 ± 1.3 mg) groups had significantly lower total morphine consumption than the control group had (16.4 ± 3.1 mg; *p* < 0.001). Notably, patients in the ESPB group had insignificantly lower morphine consumption than those in the PVB group had (*p* = 0.076). Moreover, patients in the ESPB and PVB groups had a significantly longer time to first required anaesthesia than those in the control group (7.9 ± 1.2 versus 7.5 ± 0.9 versus 2 ± 1.2 h, respectively; *p* < 0.001). The postoperative visual analog scale scores were lower in the ESPB and PVB groups than in the control group on the first 24 h after the procedure (*p* < 0.001).

**Conclusion:**

ESPB and PVB provide effective postoperative analgesia for women undergoing MRM. The ESPB appears to be as effective as the PVB.

**Trial registration:**

The study was registered before the enrolment of the first patient at the Pan African Clinical Trial Registry (www.pactr.org) database. Identification number for the registry is (PACTR202008836682092).

## Key points

- Analgesia following breast surgery is challenging because of the complicated nature of the surgery and the complex nerve supply of the breast
- Regional anaesthesia can provide adequate pain control, reduce the perioperative needs of analgesic and anaesthetic drugs, diminish postoperative nausea/vomiting, help in reducing the chance of chronic pain development, and facilitate early rehabilitation.
- The ESPB is a recently developed regional anaesthesia procedure successfully used for different types of surgical procedures including thoracic and abdominal surgeries.
- The ESPB was found to be as effective as the PVB for providing effective perioperative analgesia for women undergoing breast surgery.

## Background

Mastectomy is one of the most frequently performed surgeries for the high incidence of breast cancer. Analgesia following breast surgery is challenging because of the complicated nature of the surgery and the complex nerve supply of the breast. A recent review showed that the nerves that lead to pain vary, depending on the surgery type, and that different regional anaesthesia techniques cover different parts of the surgical field [1].

Several regional anaesthesia techniques have recently evolved. Pectoralis blocks (PECS 1 and 2) and serratus anterior plane blocks have been successfully used for perioperative analgesia following breast surgeries [2].

Regional anaesthesia can provide adequate pain control, reduce the perioperative needs of analgesic and anaesthetic drugs, diminish postoperative nausea/vomiting (PONV), help in reducing the chance of chronic pain development, and facilitate early rehabilitation [3].

PVB has been proved to be one of the most effective regional anaesthesia techniques for effective postoperative analgesia [4]. However, this is also a particularly challenging technique because of the anatomic proximity of the pleura and central neuraxial system [5]. PVB is characterized by blocking several dermatomes achieving various beneficial effects like adequate perioperative pain control, improved postoperative pulmonary functions, decreased recurrence of malignancy, and reduced risk of thrombotic disorders [6].

Several studies have reported a decrease in both postoperative pain and PONV among patients receiving PVB. Conveney et al. showed that 20% of patients with PVB required medications for PONV compared with 39% of patients with general anaesthesia (GA). This group also showed a significant decrease in the amount of postoperative opioid analgesic requirements among the PVB group (25%) compared with that among the GA group (98%) [7, 8].

The erector spinae plane block (ESPB) is a recently developed regional anaesthesia procedure successfully used for different types of surgical procedures including thoracic and abdominal surgeries. In this technique, a local anaesthetic (LA) solution is injected deep into the erector spinae muscle (ESM) with an expected paravertebral spread in both cranial and caudal directions [9].

The injected LA crosses the costotransverse foramina and blocks the ventral and dorsal rami as well as the sympathetic fibers of the corresponding spinal nerves, causing sensory blockade over the anterolateral part of the thorax. The dermatomes covered by ESPB depend on the point of entry, amount, and concentration of LA used [9, 10].

This randomized, controlled study aims to assess the analgesic effect of ESPB in patients scheduled for elective breast surgery in comparison with the well-established paravertebral block. The primary objective was to compare total morphine consumption among groups at the end of postoperative 24 h. Furthermore, intraoperative analgesic consumption, intraoperative hemodynamic response to surgical stimulation, postoperative numerical visual analog scale (VAS) scores, and incidence of PONV were the secondary objectives.

## Methods

This study is a randomized, double-blind controlled clinical trial conducted with 90 patients scheduled for a modified radical mastectomy due to breast cancer. Patients and outcome assessors were blinded to the study group. This study adheres to the applicable EQUATOR guidelines (www.consort-statement.org) and was approved by the Institutional Review Board of Suez Canal University (research #4196) (Chairperson: Professor Amani Waheed) on July 13, 2020, and was registered before enrolment of the first participant to the PACTR (www.pactr.org) database (PACTR202008836682092; date of registration: 14/8/ 2020). This study was performed during the period from September 2020 to June 2021. The patients were randomly assigned to one of the three groups using a computer-generated software program (http://www.randomizer.org),done by assistant anesthesiologist, after obtaining informed written consent from all patients.Group 1: ESPB group (30 patients), received ESPB after GA, Group 2: PVB group (30 patients), received PVB after GA and Group 3: Control group (30 patients), received GA and 30 mL of 0.9% saline injected either in the PV space or ESP. The allocation sequence was concealed using sealed opaque envelopes. The inclusion criteria included American Society of Anesthesiologists (ASA) I or ASA II patients, aged 20–60 years, and scheduled for MRM. Patients who have known allergy to LAs, body mass index (BMI) > 35 kg**.**m^**−**2^, heart block greater than first degree, renal or hepatic dysfunction, or underlying coagulopathies or those who refused to participate in the study were excluded.

### Preoperative assessment

During the preoperative visit, the procedure was fully explained to patients including the benefits and expected complications. Medical history taking was done for review of any chronic medical disorders, history of previous surgeries, and anaesthetic history with impact on any previous perioperative complications that could be related to anaesthesia. Physical examination included general examination; heart, chest, and abdominal examinations; and airway assessment. Laboratory investigations included complete blood count, coagulation profile, renal function test, liver function test, and random blood sugar. Patients received adequate training on the day before surgery for using the electrical Patient-controlled analgesia (PCA) machine. Patients fasted for 6–8 h. Moreover, midazolam (7.5 mg) was administered via oral route 60 min before the entrance to the operating theater with a little amount of water.

### Intraoperative management

Monitoring equipment (Datex-Ohmeda™, GE Healthcare Systems, Louisville, KY, USA) were used including electrocardiogram, noninvasive blood pressure, pulse oximeter, and capnography. The depth of anaesthesia was monitored with bispectral index (BIS™ Covidien, Dublin, Ireland). The target BIS range was approximately 50 for surgical anaesthesia. 0.9% saline (10 mL kg^-1^) was given to all patients 10–15 min before anaesthesia induction. After preoxygenation with 100% oxygen for at least 3 min, anaesthesia induction was started using intravenous (I.V.) fentanyl (2 mcg kg^−1^), propofol (2 mg kg^−1^), and cisatracurium (0.15 mg kg^−1^). Maintenance of anaesthesia was carried out through a closed anesthesia circuit by 1–2 MACs of isoflurane in 2L of 50% oxygen and air mixture to keep BIS in the range of 40–60, and cisatracurium (0.03 mg kg^−1^) guided by neuromuscular monitoring. The patient is turned lateral after endotracheal intubation, and regional anaesthetic technique is commenced. Inadequate analgesia in the form of increased mean arterial pressure (MAP) or heart rate of > 25% of baseline measures on two successive readings was managed by I.V. fentanyl (0.5 mcg kg^−1^). All patients received I.V. paracetamol (1 gm) and ondansetron (4 mg) 30 min before the end of the surgery. After recovery from anaesthesia and in the postanaesthesia care unit, an analgesic regimen, comprising I.V. patient-controlled morphine analgesia (1 mg bolus, 10 min lockout, and 5 mg h^−1^ maximum dose) was started. Moreover, ketorolac (30 mg I.V.) every 12 h alternating with paracetamol (1 mg I.V.) every 12 h for 48 h was used in all groups.

### ESPB technique

The patient was placed in the lateral position, and the T3 spinous process was then identified by counting down from the C7 spinous process. A linear array high-frequency ultrasonography (US) probe (Sonoite M-Turbo, Bothell, WA, USA) was positioned in the midline in a craniocaudal orientation and slid laterally to identify the T4 transverse process, ESM, rhomboid major, and trapezius muscle. Under complete aseptic precautions and after skin infiltration with a LA, a 10 cm block needle (Stimuplex® Ultra 360® 22 G, B-Braun, Melsungen, Germany) was introduced in-plane craniocaudally and navigated until reaching the TP. Under real-time US guidance, 30 mL of 0.25% bupivacaine was injected deep into the ESM with drug observation craniocaudally spread.

### PVB technique

The patient was placed in the lateral position, and the superior aspect of the fourth spinous process was then marked. The ultrasound transducer was applied in the para-median sagittal plane approximately 2.5 cm lateral to the midline till identification of the paravertebral space (PVS). Under complete aseptic precautions and after skin infiltration with a LA, a 10 cm block needle (Stimuplex® Ultra 360® 22 G, B-Braun) was inserted in a cranial to caudal direction targeting the PVS. After perforating the costotransverse ligament and negative aspiration for blood, air, or spinal fluid, 30 mL of 0.25% bupivacaine was injected under real-time US guidance superficial to the pleural line. Displacement of the pleura line anteriorly confirmed proper injection of the local anaesthetic solution.

### Measurements

Intraoperative heart rate and blood pressure were measured every 5 min for the first 30 min after induction of anaesthesia and then every 15 min until the end of the surgery. Consequently, the total intraoperative fentanyl and isoflurane consumption were recorded. Isoflurane consumption was measured by using data extracted from a modern gas analyzer included in the anesthesia machine. The first request of postoperative analgesia; total morphine consumption during the first 24 h postoperative by PCA; VAS at postoperative 1, 4, 8, 12, and 24 h; and postoperative adverse events (e.g., PONV, regional block-related hematoma formation, and pneumothorax) were recorded.

### Statistical analysis

A sample size of 14 patients per group was required to detect 9.32 mg differences between the means of 24 h postoperative morphine consumption between the ESPB and control groups at a standard deviation of 7.44 [11] with 90% power and a 5% level of significance. To account for expected dropouts, this study was performed on 30 patients for each group.

Retrieved data were summarized and processed with IBM SPSS statistical software (version 22; IBM, Armonk, NY, USA) for the Windows 10 operating system. Age, surgery duration, intraoperative heart rate, intraoperative MAP, total postoperative opioid consumption, total intraoperative fentanyl and isoflurane consumption, time to first required analgesic, and VAS during the first postoperative 24 h were summarized, according to normality, into mean (± standard deviation [SD]) or median (range). According to data normality, the hypothesis of significant differences between the two studied groups was challenged using the one-way analysis of variance (with least significant difference correction) or Kruskal–Wallis tests (VAS). Moreover, a *p* value of < 0.05 was regarded to be statistically significant.

## Results

**Fig. 1:**
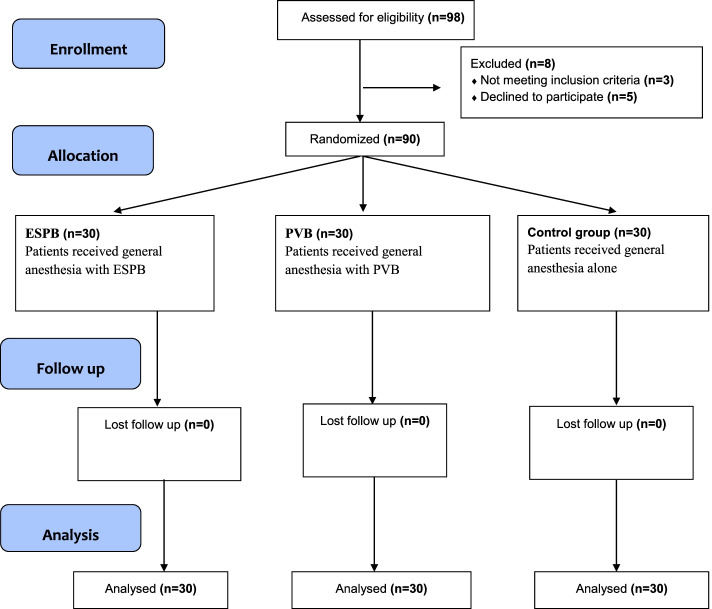
Flowchart of patient’s participation throughout the study

The present trial includes 90 patients (30 patients per group) (Fig. 1). The mean age of the patients was comparable across the three studied groups (*p* = 0.68). Similarly, the mean BMI, ASA status, comorbidities (HTN,DM), and the duration of surgery did not differ significantly across the studied groups (*p* = 0.57,0.133,0.42,0.63 and 0.41, respectively; Table 1).

**Table 1:** Clinical characteristics of the studied groups

**Variables**		**ESPB (*n* = 30)**	**PVB (*n* = 30)**	**Control (*n* = 30)**	***P*-value**
**Age (years),** mean ± SD		44.9 ± 8.1	46.6 ± 7.9	46.4 ± 8.3	0.68
**BMI,** mean ± SD		29.3 ± 3.3	28.7 ± 3.5	28.4 ± 3.4	0.57
**Duration of surgery (hours),** mean ± SD		2.3 ± 0.3	2.3 ± 0.4	2.2 ± 0.3	0.41
**ASA status**	**I**	23 (76.7%)	24 (80%)	24 (80%)	0.133
	**II**	7 (23.3%)	6 (20%)	6 (20%)	
**HTN, No. (%)**		6 (20%)	3 (10%)	3 (10%)	0.42
**DM, No. (%)**		2 (6.7%)	4 (13.3%)	4 (13.3%)	0.63

*ASA*: American Society of Anesthesiologists; *BMI*: Body mass index; *DM*: Diabetes mellitus; *HTN*: hypertension

*p:* *p* – value for comparing between the study groups

^*^:Statistically significant at *p* ≤ 0.05

**Fig. 2:**
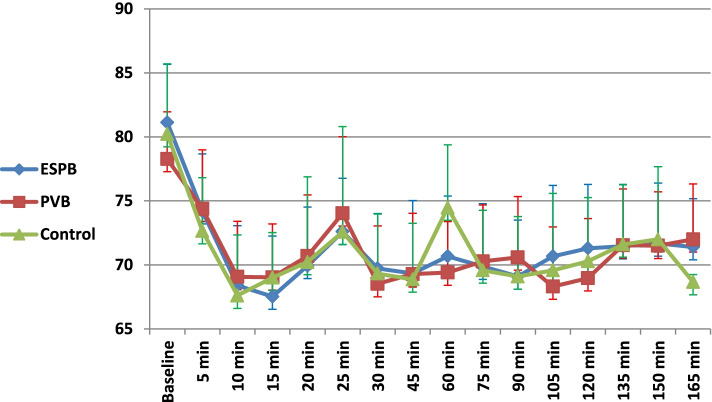
Change in the heart rate over the study procedure

**Fig. 3:**
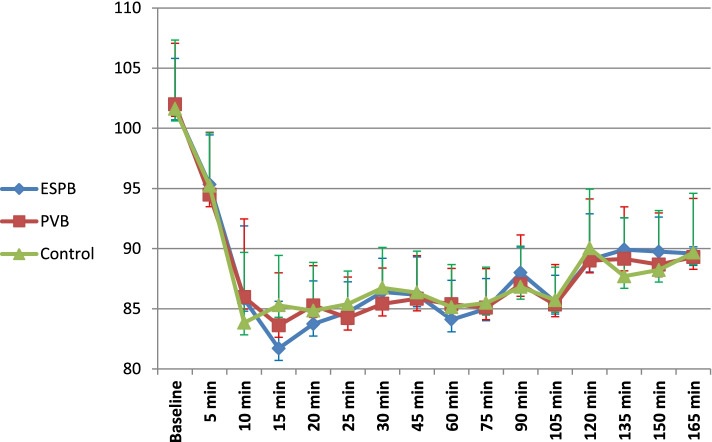
Change in the mean arterial blood pressure over the study procedure

The mean heart rate did not differ significantly across the studied groups at baseline (*p* = 0.06), 5 (*p* = 0.24), 10 (*p* = 0.47), 15 (*p* = 0.28), 20 (*p* = 0.86), 25 (*p* = 0.6), 30 (*p* = 0.57), 45 (*p* = 0.92), 75 (*p* = 0.84), 90 (*p* = 0.36), 105 (*p* = 0.25), and 120 (*p* = 0.18) min during the operation. However, the mean heart rate was significantly lower in the ESPB (70.7 ± 7.4 bpm) and PVB (69.4 ± 3.9 bpm) groups than in the control group (74.5 ± 4.9 bpm) at the 60 min of the operation (*p* = 0.002 and 0.001, respectively)( Fig. 2). The MAP did not differ significantly across the studied groups at baseline (*p* = 0.95), 5 (*p* = 0.74), 10 (*p* = 0.32), 20 (*p* = 0.25), 25 (*p* = 0.31), 30 (*p* = 0.22), 45 (*p* = 0.83), 60 (*p* = 0.28), 75 (*p* = 0.79), 90 (*p* = 0.33), 105 (*p* = 0.87), and 120 (*p* = 0.62) min during the operation. However, MAP was significantly lower in the ESPB group than the control group at 15 min of the operation (81.7 ± 3.9 versus 85.3 ± 4.2 mmHg, respectively; *p* = 0.001) (Fig. 3).

Total intraoperative fentanyl consumption was statistically higher in the control group (2.7 ± 0.5 mcg kg ^-1^) than in both the ESPB group (1.1 ± 0.2 mcg kg ^-1^; *p* < 0.001) and the PVB group (1.1 ± 0.2 mcg kg^-1^; *p* < 0.001), with no statistically significant difference between both ESPB and PVB groups (*p* = 0.45) (Table 2).

**Table 2:** Clinical outcomes of the studied groups

Variables	ESPB(I) (*n* = 30)	PVB(II) (*n* = 30)	Control(III) (*n* = 30)	*P*—value	I vs. II	I vs. III	II vs. III
**Total morphine consumption (mg),** mean ± SD	4.9 ± 1.2	5.8 ± 1.3	16.4 ± 3.1	< 0.001*	0.076	< 0.001*	< 0.001*
**Time for 1**^**st**^** required analgesia (hours),** mean ± SD	7.9 ± 1.2	7.5 ± 0.9	2 ± 1.2	< 0.001*	0.24	< 0.001*	< 0.001*
**Intraoperative fentanyl (mcg Kg**^**-1**^**),** mean ± SD	1.1 ± 0.2	1.1 ± 0.2	2.7 ± 0.5	< 0.001*	0.45	< 0.001*	< 0.001*
**Intraoperative isoflurane (ml),** mean ± SD	7.9 ± 0.8	8.2 ± 0.8	13.6 ± 1.9	< 0.001*	0.18	< 0.001*	< 0.001*
**VAS at 1 h,** median (IQR)	1 (1 -2)	1 (1–2)	2 (2 -5)	< 0.001*	0.71	< 0.001*	< 0.001*
**VAS at 4 h,** median (IQR)	2 (1.75 – 3)	2 (2 – 3)	5 (4– 6)	< 0.001*	0.99	< 0.001*	< 0.001*
**VAS at 8 h,** median (IQR)	4 (3 -5)	5 (4.5 -6)	6 (5 -7)	< 0.001*	0.001*	< 0.001*	< 0.001*
**VAS at 12 h,** median (IQR)	5 (4.75 – 6)	6 (5 – 6.5)	6 (6 – 7)	< 0.001*	0.002*	< 0.001*	< 0.001*
**VAS at 24 h,** median (IQR)	5 (4 -5)	5 (4.5 -6)	6 (5 -7)	< 0.001*	0.44	< 0.001*	< 0.001*
**PONV, N (%)**	3 (10%)	3 (10%)	6 (20%)	0.42	–-	–-	–-

I vs. II ESPB vs. PVB;I vs III: ESPB vs. Control; II vs. III: PVB vs. control

*p*:*p* – value for comparing between the study groups

^*^: Statistically significant at *p* ≤ 0.05

Concerning total morphine consumption, the ESPB (4.9 ± 1.2 mg) and PVB (5.8 ± 1.3 mg) groups had significantly lower total morphine consumption than the control group (16.4 ± 3.1 mg; *p* < 0.001). Notably, patients in the ESPB group had insignificantly lower morphine consumption than the PVB group (*p* = 0.076). Patients in the ESPB and PVB groups had a significantly longer time to first required analgesia than the control group (7.9 ± 1.2 versus 7.5 ± 0.9 versus 2 ± 1.2 h, respectively; *p* < 0.001). However, the difference between the ESPB and PVB groups was not statistically significant (*p* = 0.24). Concerning postoperative pain, the VAS scores were consistently lower in the ESPB and PVB groups than the control group on the first 24 h after the procedure (*p* < 0.001). The ESPB had an extended analgesic effect than the PVB as indicated by the significant differences in the VAS score at 8 h (median = 5 (4.75–6) versus 6 (5–6.5), respectively; *p* = 0.001) and 12 h (median = 5 (4–5) versus 5 (4.5–6), respectively; *p* = 0.002) after the operation (Table 2). The PONV incidence was numerically lower in the ESPB and PVB groups (10% each) than in the control group (20%). However, this difference did not reach the level of statistical significance (*p* = 0.42).

## Discussion

A plethora of preemptive analgesic modalities was noted for the management of postoperative pain among women undergoing breast surgery. Nonetheless, recent reports still highlight a significant postoperative pain burden in this population [12].

Breast surgeries are burdened with a high incidence of acute postoperative pain. The current body of evidence demonstrates that the inadequate management of acute postoperative pain significantly increases the risk of in-hospital mortality, functional impairments, and chronic pain [13]. Although the protocols for the management of postoperative pain vary substantially among different centers, postoperative morphine forms the basis for the universal management of moderate-to-severe postoperative pain [14]. However, opioids are generally associated with a dose-dependent increase in the risk of side effects. Such side effects can range from mild PONV to severe respiratory depression and mortality [15]. Regional anaesthesia has recently gained increased popularity as an effective preemptive approach for patients undergoing breast surgery [16]. The present trial demonstrated that the regional anaesthesia techniques, ESPB and PVB, prolonged postoperative analgesia duration and reduced morphine consumption during the first 24 h after the operation. The findings of the present study come in line with several published trials demonstrating effective analgesia following ESPB or PVB among women undergoing breast surgery [17–20].

Gürkan et al. [21] showed that ESPB significantly reduces postoperative morphine consumption by 65% among women undergoing MRM. The ESPB was effective in improving the quality of recovery scores and reducing the VAS scores among women undergoing MRM in Yao et al. [22]. Another recent single-center trial from India showed similar findings [23]. Such findings were confirmed by recent systematic reviews assessing ESPB in women undergoing breast surgery [24, 25]. Moreover, the analgesic efficacy of the conventional technique, the PVB, appears to be established by a large number of randomized trials on women undergoing breast surgery [26].

The present trial noted that the ESPB was as effective as the PVB in reducing postoperative morphine consumption and postoperative pain at the end of the first postoperative day. The equal analgesic effect of ESPB to the PVB potentially stems from its ease of performance with no major technical difficulties compared with the PVB. The widespread cutaneous sensory block by the ESPB may represent another mechanistic explanation of the present findings [27, 28]. Similarly, other reports showed similar opioid-sparing effects between PVB and ESPB in women undergoing breast surgery. For example, a previous trial by El Ghamry and Amer [29] demonstrated no significant differences between PVB and ESPB regarding the amount of postoperative morphine consumption and pain among women undergoing MRM. The same observations were reported by Moustafa et al. [30] in which ESPB and PVB exhibited no significant differences in the opioid-sparing effects among women undergoing MRM. Another 2017 randomized controlled trial reported similar findings [11]. In a recent systematic review and meta-analysis, the pooled effect estimates demonstrated no significant differences between the ESPB and PVB in terms of postoperative analgesia among women undergoing breast surgery [26].

Nevertheless, the findings of the present study come in contrast to a recent randomized, double-blind trial by Swisher et al. [31], which demonstrated a superior postoperative analgesic effect of PVB over the ESPB in women undergoing nonmastectomy breast surgery. The PVB resulted in lower morphine consumption and VAS scores than the ESPB in the first 24 h after surgery. The authors hypothesized that the superior analgesic effect of PVB may stem from the insufficient spread of the LAs to the paravertebral space following ESPB compared with the direct spread of the LAs to the paravertebral space following PVB [32, 33].

The present study hypothesized that the current inconsistency in the results of published literature can be attributed to a myriad of reasons. First, the utilization of various concentrations of LAs can alter the analgesic effect of various nerve block techniques. Second, the concentration and volume of LAs were reported to play role in the extent of dermatomes covered by ESPB [10]. Third, the level of the operator experience plays a critical role in the success and level of anaesthesia of the used nerve block technique. The PVB poses a technical difficulty due to the surrounding anatomical structures, especially the pleura and central neuraxial system [5]. Lastly, the various surgical procedures and techniques may further explain the inconsistency in the results of published literature. Despite this level of uncertainty in the published literature, it can be empirically concluded that the ESPB is an effective analgesic modality as the PVB and, hence, it can be utilized in low-resourced facilities or, in which, the anesthesiologists have less experience with PVB.

As previously mentioned, the main advantage of nerve block techniques lays in their ability to reduce postoperative opioid consumption and the subsequent risk of complications (e.g., PONV and respiratory depression) [15]. The present report noted that both ESPB and PVB reduced the PONV incidence in comparison with GA alone. Such findings are in line with previous randomized controlled trials [17–26]. Moreover, PVB carries the risk of serious complications due to its anatomic proximity to critical structures (e.g., pneumothorax) [34]. The present study did not observe the occurrence of any technique-related adverse events.

It is believed that only a few trials have compared the efficacy of ultrasound-guided ESPB and PVB in women undergoing breast surgery. The predetermined calculation of sample size, proper randomization of the patients, and the utilization of double-blind design are among the strengths of this trial. The same anesthesiologist conducted all procedures to avoid performance bias, which is an additional strength. However, the present study acknowledged the existence of certain limitations. The lack of pain assessment during the movement of the patients and the use of single injection to perform the nerve block, rather than a catheter, are among the study’s limitations. Another limitation is the single-center nature of this trial. Finally, we did not use ranitidine as a premedication in the current study because it was withdrawn from our institute after the USFDA warning about its safety.

## Conclusion

Ultrasound-guided ESPB and PVB provide effective postoperative analgesia for women undergoing MRM when compared with GA alone. This double-blind study showed that both techniques provided superior analgesia and lower total morphine consumption when compared with GA only.

## Data Availability

The datasets generated during and/or analysed during the current study are available from the corresponding author on reasonable request.

## Abbreviations

ESPB: Erector spinae plane block
MRM: Modified radical mastectomy
PVB: Paravertebral block
PECS: Pectoralis blocks
PONV: Postoperative nausea/vomiting
GA: General anaesthesia
LA: Local anaesthetic
ESM: The erector spinae muscle
VAS: Visual analog scale
BMI: Body mass index
BIS: Bispectral index
MAP: Mean arterial pressure
PCA: Patient-controlled analgesia
US: Ultrasonography
TP: Transverse process
USFDA: United States Food and Drug Administration
